# Control of hypertension in the critically ill: a pathophysiological approach

**DOI:** 10.1186/2110-5820-3-17

**Published:** 2013-06-27

**Authors:** Diamantino Ribeiro Salgado, Eliezer Silva, Jean-Louis Vincent

**Affiliations:** 1 Department of Intensive Care, Erasme Hospital, Université Libre de Bruxelles, route de Lennik 808, Brussels 1070, Belgium; 2 Dept of Internal Medicine, Universidade Federal do Rio de Janeiro, Rua Professor Rodolpho Paulo Rocco, 255 Sala 4A, Rio de Janeiro 12-21941-913, Brazil; 3 Intensive Care Unit, Albert Einstein Hospital, Sao Paulo, Brazil

**Keywords:** Beta-blockers, Calcium channel blockers, Cardiac output, Diuretics, Mean arterial pressure, Vasodilators

## Abstract

Severe acute arterial hypertension can be associated with significant morbidity and mortality. After excluding a reversible etiology, choice of therapeutic intervention should be based on evaluation of a number of factors, such as age, comorbidities, and other ongoing therapies. A rational pathophysiological approach should then be applied that integrates the effects of the drug on blood volume, vascular tone, and other determinants of cardiac output. Vasodilators, calcium channel blockers, and beta-blocking agents can all decrease arterial pressure but by totally different modes of action, which may be appropriate or contraindicated in individual patients. There is no preferred agent for all situations, although some drugs may have a more attractive profile than others, with rapid onset action, short half-life, and fewer adverse reactions. In this review, we focus on the main mechanisms underlying severe hypertension in the critically ill and how using a pathophysiological approach can help the intensivist decide on treatment options.

## Introduction

Arterial hypertension is a worldwide problem, affecting more than 1 billion people [1,2]. Chronic arterial hypertension is an important cardiovascular risk factor and associated with significant morbidity and mortality in the general population [3]. Chronic hypertension also is the primary risk factor for cerebrovascular disease [4]. Acute hypertension is not uncommon in the emergency room or acute care setting [5] and can have important consequences on various organs, including the heart [6], the kidneys [7], the brain, and the lungs; associated end-organ injury has been reported in 19-81% of patients with acute severe hypertension [8-10]. In a recent American survey of patients with severe acute hypertension (defined as systolic arterial pressure [SAP] >180 mmHg and/or diastolic arterial pressure [DAP] >110 mmHg) requiring hospitalization, new or worsening end-organ dysfunction was observed in 59% of subjects [10]; the 90-day mortality rate was 11% [10]. More than one third of patients discharged home were rehospitalized at least once within 90 days, 29% for acute severe hypertension [11]. Interestingly, 28% of the patients had a primary neurological diagnosis and mortality rates were higher in these patients than in those without a neurological diagnosis (24% vs. 6%, *p* < 0.0001) [12].

According to general guidelines [1], moderate arterial hypertension is defined as a systolic arterial pressure (SAP) ≥140 mmHg or a diastolic arterial pressure (DAP) ≥90 mmHg, also known as Stage I hypertension. This degree of hypertension is rarely treated in critically ill subjects, unless it is accompanied by obvious harmful hemodynamic symptoms. Stage II hypertension (systolic arterial pressure [SAP] >160 mmHg or diastolic arterial pressure [DAP] >100 mmHg [1]) is more frequently associated with acute cardiovascular complications, including acute heart failure, intensive care unit (ICU) admission, prolonged hospital stay, and death [1,10,12,13]. Values of SAP >180 mmHg and/or DAP >110 mmHg often are used to define severe hypertension [10,14].

In discussing hypertension, it is important to differentiate some terms. Hypertensive *emergencies* are defined as a marked increase in arterial pressure associated with acute, life-threatening target-organ injuries (Table 1), often requiring hospitalization in an ICU for immediate pressure control. Hypertensive *urgencies* are not associated with imminent acute end-organ injury, so that blood pressure control can be slower, over several hours, and hospitalization may not even be necessary [1]. Because there is no consensus regarding the cutoff value of blood pressure for defining hypertensive urgencies, diagnosis should be individualized after taking into account several factors, such as age, sex, the presence of chronic hypertension (and use of antihypertensive drugs), and the presence of comorbidities. Indeed, the same degree of arterial hypertension may be associated with symptoms of acute target-organ damage in one individual or be completely asymptomatic in another. Nevertheless, in general terms, a blood pressure equal to or greater than 180/100 mmHg may require intervention [15].

**Table 1:** Most frequent target organs damaged during acute hypertensive crises

**Organ system**	**Example of Injury**
Cardiovascular	Acute coronary syndromes
	Heart failure and pulmonary edema
	Aortic dissection
Central nervous system	Stroke or transient ischemic attack
	Acute encephalopathy/cerebral edema
	Retinal hemorrhage
Renal	Acute renal failure

Before initiating therapy for severe acute hypertension in a critically ill patient, the presence of precipitating causes should be looked for (Table 2), because removal and/or control of these factors can prevent unnecessary treatment in many instances. In the patient with chronic hypertension, it may be wise to continue the antihypertensive medications. Unlike some other traditional reviews that cover the management of hypertensive urgencies/emergencies in specific diseases (e.g., acute aortic dissection, preeclampsia, cerebrovascular accident) [16-18], we focus on the main mechanisms underlying severe hypertension in the critically ill and how a pathophysiological approach can help the practicing intensivist to manage this problem.

**Table 2:** Principal causes of acute systemic arterial hypertension in critically ill patients

**Organ system**	**Cause**
**Decompensated essential (primary) hypertension Secondary causes**	Discontinuation of antihypertensive drugs
Central Nervous System	Pain
	Anxiety and stress
	Delirium
	Withdrawal syndromes
	Intracranial hypertension
Renal system	Urinary retention
	Renal failure
	Hypervolemia
Respiratory system	Respiratory distress – Hypoxemia, hypercapnia
Metabolic	Hypoglycemia
	Steroid administration
	Pheochromocytoma
	Cushing syndrome
	Intoxication, substance abuse and overdose (cocaine, phencyclidine, amphetamines)

## Review

### Pathophysiology of arterial hypertension

Regional blood flow and organ perfusion are determined by the driving pressure and by vascular autoregulation, a functional property of vessels that permits blood flow to adapt to a given level of arterial pressure and tissue metabolic demand [19,20], thus protecting tissues against the negative effects of excessive or insufficient flow observed during hypertension and hypotension, respectively. In healthy subjects, for example, cerebral blood flow is kept relatively constant through a wide range of arterial pressures (usually a mean arterial pressure [MAP] from 60-120 mmHg) [5]. Various mechanisms are involved in vessel autoregulation, including activation of the autonomic system and local production of vasoactive substances, such as angiotensin II, endothelin, prostanoids, nitric oxide (NO), adenosine, reactive oxygen species (ROS), and lactate [21,22]. Endothelial dysfunction also may contribute to the development of systemic hypertension. In this respect, preeclampsia, a disease model of systemic endothelial dysfunction, and some recent chemotherapy drugs that inhibit vascular endothelial growth factor (VEGF), share similar physiological mechanisms for the genesis of hypertension by inhibiting VEGF, reducing the production of NO and increasing systemic vascular resistance (SVR) [23].

When faced with a patient with severe acute hypertension, one needs to keep in mind the two determinants of arterial pressure [24]:

(1) MAP=CO×SVR

where CO is the cardiac output. SVR essentially represents the degree of vascular tone. Because CO is the product of the heart rate (HR) and the stroke volume (SV), we can write:

(2) $$\mathrm{MAP}=\left(\mathrm{HR}\;\times \;\mathrm{SV}\right)\times \;\mathrm{SRV}$$

The vascular tone (SVR) is mainly determined by the degree of constriction of precapillary and small arterioles and, to a lesser degree, by the blood viscosity (k). The vascular tone is an important determinant of ventricular afterload. Cardiac output is determined by heart rate, afterload, myocardial contractility, and preload. Preload is largely determined by the volume status, which is represented by the central venous pressure (CVP), pulmonary artery occlusion pressure (PAOP), or inferior vena cava diameter in the clinical setting. Cardiac contractility is difficult to assess at the bedside but can be indirectly appreciated by the assessment of the ventricular stroke work index (basically derived from the product of stroke index and MAP) and the ejection fraction obtained by echocardiography [25]. Increased myocardial contractility can, in principle, contribute to the development of hypertension, although this would only be observed in hyperadrenergic conditions.

Combining all these aspects, a final equation for arterial pressure can be derived:

(3) $$\mathrm{MAP}=\left(\mathrm{HR}\;\times \;\left[\mathrm{preload}\;x\;\mathrm{myocardial}\;\mathrm{contractility}\right]\right)\;\times \;\mathrm{SVR}$$

Physicians will be able to manage most cases of severe hypertension in critically ill patients by associating these physiological principles with knowledge of the pharmacological properties of the available antihypertensive drugs.

### Treatment options for severe acute hypertension inthe ICU

In patients with chronically *uncontrolled* hypertension, the circulation in vital organs, such as the brain, heart, and kidney, adapts, resulting in arteriolar hypertrophy. These patients are prone to develop organ ischemia when blood pressure is rapidly reduced, even to levels considered as relatively high in normotensive patients [5,26], and one should be particularly cautious when controlling arterial pressure in such individuals. As a general rule for the treatment of hypertensive emergencies, one should not try to reduce the MAP by more than 20% (or diastolic blood pressure by 10-15% or to approximately 110 mmHg) during the first hour, except in acute aortic dissection where this goal should be achieved within 10 min [27].

There are a large number of therapeutic options for severe hypertension. We will restrict our discussion to the agents most frequently used in ICU patients (Tables 3 and 4).

**Table 3:** Main cardiovascular effects of the different antihypertensive drugs

	**Heart rate**	**Myocardial contractility**	**Preload**	**Systemic vascular resistance**
*Nitrates*				
Nitroglycerin	+	0	_ _	_
Nitroprusside	+	0	_ _	_ _
*Hydralazine*	+/++	0	0/+	_ _ _
*ACE inhibitors and AT1 receptor blockers*	0	0	_	_ _ _
*Calcium channel blockers*				
Amlodipine	_	_	0	++
Nifedipine	_	_	0	+ + +
Nicardipine	0	_	0	+++
Diltiazem	_ _	_	0	+
*β−blockers*	_ _ _	_ _	0	+
β-blockers with intrinsic	_	_	0	0/_
sympathomimetic activity				
*Phentolamine*	0/ _	_	0/+	_ _ _
*Urapidil*	0/+	0	_ _	_ _ _
*Diuretics*	0/+	0	_ _ _	+ +
*Clonidine*	_ _	0	0	_ _
*Methyldopa*	0/ _	0/ _	0/ +	_ _

0, no effect; - Slight negative effect; -- moderate negative effect; --- strong negative effect.

+ Slight positive effect; + + moderate positive effect; + + + strong positive effect.

*ACE* angiotensin-converting enzyme, *AT1* angiotensin II subtype 1.

**Table 4:** **Doses**, **pharmacokinetics**, **and pharmacodynamics of the intravenous antihypertensive agents most frequently used in intensive care units**

**Drugs**	**Intravenous dose**	**Onset/peak of action**	**Half**-**life/duration of action**	**Metabolism/excretion**	**Main side effects**	**Main clinical indications**
**Sympatholytic drugs**						
*β-blockers*						
Propranolol (β_1_- and β_2_-receptor blocker)	1-3 mg every 2–5 min (over 1–30 min)	Onset: 5 min	Half-life: 3–5 h	Metabolism: hepatic CYP450	Hypotension, heart failure, heart block, dizziness, fatigue, confusion, depression, bronchospasm, Raynaud´s phenomenon, diarrhea, pruritus, rash	Cardiac ischemic syndromes with arterial hypertension and normal heart function. Avoid in pheochromocytoma crises
	Max dose: 5 mg		Duration: 6–12 h	Excretion: urine (96-99%)		
	Infusion: not recommended					
Metoprolol (selective β_1_-receptor blocker)	5 mg every 3 min (over 1–30 min)	Onset: 5–10 min	Half-life: 3–7 h	Metabolism: hepatic CYP2D6	Hypotension, heart failure, heart block, dizziness, fatigue, depression, bronchospasm, diarrhea, pruritus, rash	Cardiac ischemic syndromes with arterial hypertension and normal heart function
	Max dose: 15 mg		Duration: 5–8 h	Excretion: urine (5-10% unchanged)		
	Infusion: not recommended					
Labetalol (α_1_, β_1_, and β_2_-receptor blocker)	Bolus: 20–80 mg every 10 min. Max dose: 300 mg	Onset: 5–10 min	Half-life: 6 h	Metabolism: hepatic glucuronide conjugation	Nausea, scalp tingling, bronchospasm, dizziness, heart block, orthostatic hypotension	Most hypertensive emergencies, good for hypertension in neurocritically ill patients and pregnancy; caution in heart failure
	Infusion: 0.5-2.0 mg/min	Peak: 5–15 min	Duration: 3–18 h	Excretion: urine 50% (<5% unchanged), feces 50%		
Esmolol (selective β_1_-receptor blocker)	Bolus: 500 *μ*g/Kg (over 1 min)	Onset: 1–2 min	Half-life: 9 min	Metabolism: plasma esterases	Arterial hypotension, bronchospasm, heart block, heart failure	Hypertensive emergencies with normal or high cardiac output, and aortic dissection in particular. Contraindicated in pheochromocytoma crisis
	Max dose: 300 μg/Kg/min	Peak: 6–10 min	Duration: 10–30 min	Excretion: urine 70-90% (<1% unchanged)		
	Infusion: 50–300 μg/Kg/min					
*α-Adrenergic antagonists*						
Phentolamine (peripheral α-receptor antagonist)	Bolus: 5–20 mg	Onset: 1–2 min	Half-life: 19 min	Metabolism: hepatic	Tachycardia, flushing, headache, orthostatic hypotension, dizziness, nasal congestion, pulmonary hypertension	Hypertensive emergencies associated with excessive catecholamine levels
	Max dose: 15 mg	Peak: 10–20 min	Duration: 15–30 min	Excretion: urine (10% unchanged)		
	Infusion: not recommended					
Urapidil (peripheral α_1_-receptor antagonist and central serotonin antagonist)	Bolus: 12.5-25 mg	Onset: 3–5 min	Half-life: 2–4.8 h	Metabolism: hepatic	Headache, hypotension, dizziness	Hypertensive emergencies in postoperative and pregnant patients
	Max dose: 50 mg	Peak: 0.5-6 h	Duration: 4–6 h	Excretion: urine (15-20% unchanged), feces 10%		
	Infusion: 5–40 mg/h					
*Centrally acting agents*						
Clonidine (central α_2_ receptor agonist)	Bolus: not recommended	Onset: 5–10 min	Half-life: 12 h	Metabolism: Hepatic CYP450	Eye and mouth dryness, sedation, erectile dysfunction, orthostatic hypotension, bradycardia, drowsiness.	Hypertensive emergencies, particularly in the context of withdrawal syndrome and pain
	Max dose: 7.2 μg/min (or 450 μg/2 h)	Peak: 2–4 h	Duration: 6–10 h	Excretion: Urine (50% unchanged), feces/bile 20%		
	Infusion: 1.2-7.2 μg/min					
Methyldopa (central α_2_ receptor agonist)	250-1000 mg every 6-8h	Onset: >1 h	Half-life: 2 h	Metabolism: Hepatic CYP450 and central adrenergic neurons	Peripheral edema, fever, depression, sedation, dry mouth, bradycardia, hepatitis, hemolytic anemia, lupus-like syndrome	Hypertensive emergencies, particularly in pregnant patients. Use limited by adverse effects
	Max dose: 1000 mg every 6h	Peak: 6–8 h	Duration: 12–24 h	Excretion: urine (85% metabolites), feces		
	Infusion: not recommended					
Dexmedetomidine (central α_2*−*_receptor agonist)	Bolus: 1 μg/Kg/min over 10 min (not necessary in sedated patients)	Onset: 6–15 min	Half-life: 2–2.5 h	Metabolism: hepatic	Hypotension, bradycardia, fever, nausea, vomiting, hypoxia, anemia	Hypertensive urgencies associated with hyperactive delirium or withdrawal syndrome
	Max dose: 1.5 μg/Kg/h	Peak: 1 h	Duration: 4 h	Excretion: urine (metabolites)		
	Infusion: 0.2-0.7 μg/Kg/h					
**Calcium channel blockers**						
Nicardipine	Bolus: not recommended	Onset: 5–10 min	Half-life: 2–4 h	Metabolism: hepatic CYP3A4	Tachycardia, headache, flushing, peripheral edema, angina, nausea, AV block, dizziness	Most hypertensive emergencies; caution in heart failure
	Max dose: 30 mg/h	Peak: 30 min	Duration: 4–6 h	Excretion: urine 60% (<1% unchanged), feces 35%		
	Infusion: 5–15 mg/h					
Clevidipine	Bolus: not recommended	Onset: 1–2 min	Half-life: 1–15 min	Metabolism: plasma and tissue esterases	Atrial fibrillation, nausea, fever, insomnia, headache, acute renal failure	All hypertensive emergencies, particularly postoperative hypertension
	Max dose: 32 mg/h	Peak: 3 min	Duration: 5–15 min	Excretion: urine 63-74%, feces 7-22%		
	Infusion: 1–2 mg/h					
Diltiazem	Bolus: 0.25 mg/Kg (over 2 min)	Onset: 2–7 min	Half-life: 3.4 h	Metabolism: hepatic CYP3A4	Bradycardia, AV block, hypotension, cardiac failure, peripheral edema, headache, constipation, hepatic toxicity	Hypertensive emergencies associated with normal heart function and tachyarrhythmia
	Max dose: 15 mg/h	Peak: 7–10 min	Duration:30 min-10 h (median 7 h)	Excretion: bile, urine (2-4% unchanged)		
	Infusion: 5–15 mg/h (≤24 h)					
**ACE inhibitor**						
Enalaprilat	Bolus: 1.25-5 mg every 6 h	Onset: 15–30 min	Half-life: 11 h	Metabolism: hepatic biotransformation	Hypotension, headache, worsening of renal function, hyperkalemia, angioedema, cough, agranulocytosis	Hypertensive emergencies associated with left ventricular dysfunction; caution in hypovolemia
	Max dose: 5 mg every 6 h	Peak: 3–4.5 h	Duration: 6–12 h	Excretion: urine (60-80%)		
	Infusion: not recommended					
**Arterial vasodilators**						
Hydralazine (arteriolar vasodilator)	Bolus: 10–20 mg every 4–6 h	Onset: 5–20 min	Half-life: 2–8 h	Metabolism: Hepatic acetylation	Hypotension, tachycardia, headache, facial flushing, angina pectoris, vomiting, paradoxical hypertension, lupus-like syndrome	Hypertensive emergencies, especially severe hypertension in pregnancy
	Max dose: 40 mg per dose	Peak: 30–60 min	Duration: 1–8 h	Excretion: urine 52-90% (14% unchanged), feces 10%		
	Infusion: not recommended					
Fenoldopam (selective dopamine type 1-receptor agonist)	Bolus: not recommended	Onset: 5–10 min	Half-life: 5 min	Metabolism: Hepatic methylation	Hypotension, tachycardia, headache, nausea, facial flushing, angina, ST-T wave changes, elevated intraocular pressure	Hypertensive emergencies, especially severe hypertension in patients with acute renal failure
	Max dose: 1.6 μg/Kg/min	Peak: 15 min	Duration:30–60 min	Excretion: urine 90%, feces 10%.		
	Infusion: 0.05-1.6 μg/Kg/min					
**Mixed arterial/venous vasodilators**						
Nitroprusside (nitric oxide donor)	Bolus: not recommended	Onset: 1–2 min	Half-life: <10 min (nitroprusside), 3 days (thiocyanate)	Metabolism: erythrocytes, hepatic methylation	Hypotension, tachycardia, headache, cyanide and thiocyanide intoxication, nausea, flushing, vomiting, muscle spasm, pulmonary shunt	Hypertensive emergencies, especially aortic dissection. Caution in renal and hepatic failure
	Max dose: 10 μg/Kg/min (<1 h)	Peak: 15 min		Excretion: urine		
	Infusion: 0.25-4 μg/Kg/min		Duration: 1–10 min			
Nitroglycerin (nitric oxide donor with predominant venular action)	Bolus: not recommended	Onset: 2–5 min	Half-life: 1–3 min	Metabolism: erythrocytes, hepatic, vessel wall	Hypotension, headache, dizziness, vomiting, tachyphylaxis, methemoglobinemia	Hypertensive emergencies, especially those associated with acute coronary syndrome, volume overload, or pulmonary edema
	Max dose: 300 μg/min	Peak: 5 min	Duration: 5–10 min	Excretion: urine		
	Infusion: 5–300 μg/min					
**Diuretics**						
Furosemide (inhibits reabsorption of Na/Cl in the ascending loop of Henle)	Bolus: 20–40 mg	Onset: 5 min	Half-life: 30–60 min	Metabolism: hepatic	Hypokalemia, hypovolemia, hypotension, metabolic alkalosis, ototoxicity, thrombocytopenia, pancreatitis, interstitial nephritis, hyperglycemia, hyperuricemia	Hypertensive emergencies, especially those associated hypervolemia and/or heart failure
	Max dose: 200 mg/dose or 160 mg/h	Peak: 1–2 h	Duration: 2 h	Excretion: urine 88%, bile/feces 12%.		
	Infusion: 10–40 mg/h					
Bumetanide (inhibits reabsorption of Na/Cl in the ascending loop of Henle)	Bolus: 0.5-1 mg/dose up to 2 times/day	Onset: 2–3 min	Half-life: 1–1.5 h	Metabolism: hepatic	Hypokalemia, hypovolemia, hypotension, metabolic alkalosis, ototoxicity, thrombocytopenia, pancreatitis, interstitial nephritis, hyperglycemia, hyperuricemia	Hypertensive emergencies, especially those associated with hypervolemia and/or heart failure
	Max dose: 10 mg/day	Peak: 1–4 h	Duration:4–6 h	Excretion: urine 81%, bile 2%		
	Infusion: 0.5-2 mg/h					

*ACE* angiotensin-converting enzyme.

#### Vasodilators

##### Nitroglycerin

Although described as having antihypertensive effects, nitroglycerin causes weak direct arterial vasodilation, which is observed only with high doses (>60 μg/min intravenously) [28]. Nitroglycerin has a more profound venodilating than arteriolar effect. In the presence of hypovolemia, it may cause a decrease in venous return and CO; in these conditions, reflex tachycardia is common [27]. Nitroglycerin is indicated in severe hypertension associated with volume overload and pulmonary edema. Nitroglycerin promotes coronary vasodilation without steal syndrome [29], so that it may be used at low doses (≤60 μg/min) as an adjunct to other intravenous antihypertensive agents in patients with acute coronary syndromes [14]. Nitroglycerin (as other vasodilators) may increase pulmonary shunt and aggravate systemic hypoxemia by inhibiting pulmonary hypoxic vasoconstriction.

##### Nitroprusside

Sodium nitroprusside is a potent and short-acting purely vasodilatory agent, causing both arterial and venous vasodilation, thus reducing pre- and afterload. The rapid onset of action and short half-life mean that this drug is easily titrated, but because of its potency, speed of action, and risk of tachyphylaxis, intraarterial blood pressure monitoring is recommended [15]. The major unwanted effect if used at high doses for prolonged periods (>8 hours) is the generation of the toxic metabolites, cyanide and thiocyanide, which accumulate more rapidly in the presence of renal and hepatic failures. Nitroprusside infusion also may alter gas exchange by aggravating pulmonary shunt, increase intracranial pressure by inducing vasodilation, induce coronary steal syndrome by nonselective coronary vasodilation, and may be associated with spinal ischemia and paralysis during thoracic aortic surgery [30]. Nitroprusside is now rarely used or recommended as a first-line agent; if used, limited doses and infusion durations are preferred [15,27].

##### Hydralazine

Hydralazine is a direct arterial vasodilator without significant effects on the venous system. Its mechanisms of action are complex and involve inhibition of calcium influx into vascular smooth muscle cells, either by cell membrane hyperpolarization or by induction of cGMP [31,32]. Hydralazine increases heart rate and has a slight positive inotropic effect. Hydralazine is indicated for severe hypertension associated with increased SVR, especially in the presence of bradycardia. However, hydralazine causes a reflex increase in sympathetic tone and can worsen myocardial oxygen consumption, so should be used with caution in patients with known heart disease (e.g., heart failure, coronary artery disease, or valvular dysfunction). Fluid retention resulting from activation of the sympathetic and renin-angiotensin systems following peripheral vasodilation can be managed by adding diuretic drugs if needed. Hydralazine can have prolonged antihypertensive effects and dosage titration can be difficult [33].

Hydralazine has been used for a long-time as a first-line agent for pregnancy-induced hypertensive disorders, but there is evidence suggesting that parenteral hydralazine may be associated with more adverse outcomes, including maternal hypotension, placental abruption, fetal heart rate abnormalities, Cesarean section, stillbirth, and fetal suffering compared with labetalol and calcium channel blockers [34,35].

##### Fenoldopam

As a specific dopamine type-1 receptor agonist, fenoldopam increases renal blood flow, stimulates natriuresis and urinary output, and may improve renal function, so that it may be suitable for the treatment of severe hypertension in patients with renal failure [36]. Fenoldopam has a rapid onset of action, a relatively short half-life, no rebound effect, and no negative effects on cardiac function [37,38], although it also may be associated with mild tolerance and hypokalemia after prolonged infusions. Anaphylactic reactions have been reported in patients with known sulfite sensitivity because fenoldopam contains a metabisulfite molecule. It can increase intraocular pressure and thus also is contraindicated in patients with glaucoma. Fenoldopam is not currently available in many countries.

##### Enalaprilat

Enalaprilat is an intravenous angiotensin-converting enzyme (ACE) inhibitor that reduces peripheral arterial vasoconstriction caused by angiotensin II. Its final antihypertensive effect is highly dependent on the patient’s volume status and plasma renin activity [39]. Hence, in normo- or hypervolemic patients without increased angiotensin II levels, enalaprilat will not cause major hypotension. Conversely, in hypovolemic subjects with high angiotensin II levels, the antihypertensive effect can be significant [39]. Enalaprilat may be considered in severe hypertension associated with heart failure. Because of its unpredictable effects, long half-life, and risk of excessive hypotensive response (especially in hypovolemic hyperreninemic patients), it is not considered as a first-line agent for the treatment of acute hypertension. Enalaprilat is contraindicated in pregnancy [40].

#### Calcium channel blockers

These drugs inhibit calcium influx through voltage-sensitive L-type calcium channels in vascular smooth muscle cells inducing arteriolar vasodilation and reducing SVR. They are divided into two pharmacological classes: dihydropyridines and nondihydropyridine derivatives. The dihydropyridines (e.g., nifedipine, nicardipine, amlodipine) are the largest class, and members have greater vasodilatory potency and less negative chronotropic activity, making them relatively safe for patients with hypertension. However, they can be potentially hazardous in patients with severe heart failure because of the calcium entry blocking effects. Two agents, nicardipine and clevidipine, are available for continuous intravenous infusion for the management of arterial hypertension. Nicardipine is a second-generation dihydropyridine with high hydrosolubility, and short onset but a relatively long duration of action. Its dosage is weight-independent, easily titrated, and it promotes cerebral and coronary vasodilation [41]. Nicardipine has been used for the treatment of severe hypertension, particularly in the perioperative period [42,43], and has recently been shown to be superior to labetalol in treating acute severe hypertension in emergency department patients [44], including those with end-organ damage [45], and also in critically ill patients [46].

Clevidipine, a third-generation dihydropyridine, is an ultrashort acting agent. It is metabolized by plasma esterases and has few drug interactions, making it useful for severe hypertension in critically ill patients, particularly those with renal and/or hepatic failure [47,48]. Clevidipine exerts vascular-selective, arterial specific vasodilation, without negative effects on cardiac function, because it does not significantly affect preload and induces minimal reflex tachycardia [9,49]. Clevidipine has been shown to be more effective than other vasodilators, such as sodium nitroprusside and nitroglycerin, in the control of acute hypertension during the perioperative period of cardiac surgery [49,50]. It also has been reported to be safe and effective in the management of acute severe hypertension in patients with acute heart failure and renal dysfunction [51,52]. Because it is a fairly new agent, it is relatively expensive compared with other agents.

The nondihydropyridine derivatives, which include diltiazem and verapamil, are less potent vasodilators but with significant negative chronotropic activity, making them less commonly used as antihypertensive drugs.

#### Beta-blocking agents

β-adrenergic receptor antagonists (β-blockers) inhibit the action of norepinephrine, epinephrine, and other sympathomimetic drugs acting on β receptors. As a general rule, the greater the degree of β stimulation that occurs at a given site, the greater the observed effect of β-blockers.

β-blocking agents primarily decrease blood pressure by a reduction in cardiac output (through a reduction in both contractility and heart rate). They also can reduce renin release by the juxtaglomerular cells, resulting in less angiotensin II formation, less generation of NO [53], suppression of the central nervous sympathetic outflow, alteration of baroreceptor sensitivity, and attenuation of the peripheral pressor response to catecholamines [54]. β-blockers reduce the risk of cardiovascular disease and are of particular interest for patients with ischemic cardiomyopathy, because they reduce myocardial oxygen consumption and increase coronary blood flow toward ischemic regions [55]. β-blocking agents should not be used in isolation in catecholamine-induced severe hypertension seen with pheochromocytoma or stimulant intoxication, because the unopposed peripheral α-effect could further increase blood pressure [17,56]. Obviously, β-blocking agents must be avoided in situations where a reduction in oxygen delivery could be problematic.

Various β-blockers are available, with different degrees of specificity for β1-receptor antagonism, intrinsic sympathomimetic activity, membrane-stabilizing properties, capacity to induce vasodilation, lipid solubility, half-life, onset of action, and routes of administration [57]. β-blocker agents are classified as non-subtype receptor selective (first generation), β_1_-receptor selective (second generation), and antagonists with additional cardiovascular actions (third generation). Some examples are presented in Table 4. Of particular interest is esmolol, an ultrashort acting β1-blocker, which is rapidly metabolized by plasma esterases (safe in renal and hepatic failure) and has a short half-life. Esmolol is a good option for the treatment of severe hypertension, because it can be easily titrated and discontinued in high-risk patients or if poorly tolerated, e.g., if heart failure, atrioventricular block or bronchospasm develop [58,59]. Labetalol, an example of a third generation β-blocker, competitively inhibits α_1_ and β receptors, resulting in a decrease in blood pressure by both vasodilation and reduction of the sympathetic stimulation of the heart. Labetalol infusion is useful in the management of hypertensive crises, particularly in pregnancy-induced hypertensive emergencies because of its negligible lipid solubility and placental transfer [35], but should be used with caution in patients with hepatic failure [60].

#### Alpha-blocking agents

##### Phentolamine (peripheral α-receptor antagonist)

This peripheral α-adrenergic receptor blocker has limited use in the management of severe hypertension because of the risk of severe hypotension and other adverse effects. Phentolamine is mainly indicated for the treatment of hypertensive emergencies associated with excessive circulating catecholamines, such as in pheochromocytoma crisis or cocaine intoxication [61,62]. It is no longer available in many countries.

##### Urapidil

This drug combines selective postsynaptic α_1_-adrenergic antagonist activity with central antagonism of serotonin receptors, which gives it a potent vasodilatory effect reducing both pre- and afterload, as well as causing pulmonary and renal vasodilation, without any significant effect on cardiac function. It is relatively safe because it does not affect coronary and cerebral blood flow. Urapidil has a rapid onset of action, with good efficacy. It has been used for the treatment of severe hypertension in the postoperative period and pregnancy but is contraindicated in patients with aortic isthmus stenosis, major arteriovenous shunt, and during lactation [63].

#### Diuretics

Diuretic drugs provoke inhibition of sodium chloride reabsorption at different sites in the nephron. Because diuretics reduce sodium and water load, and consequently volemia, they are particularly useful for the treatment of hypertension associated with hypervolemic and edematous states. For blood pressure control, loop of Henle diuretics (furosemide and bumetanide) are preferred for their rapid onset of action and high potency. Furosemide may have a direct venodilating effect, although this is debated [64].

Diuretics should be used with caution in acute hypertension, because these patients are often hypovolemic. Use of ACE inhibitors or other vasodilators may abolish the increment of SVR induced by furosemide [65], and the ACE inhibitor/diuretic association can be an interesting combination for the long-term treatment of hypertensive patients.

#### Centrally acting antihypertensives

##### Clonidine

Clonidine is a centrally acting α_2_-adrenergic agonist. It is more frequently used via the oral route, but an intravenous formulation is available in some countries. It can be used as an alternative to β−blocking agents, because it associates some vasodilating properties with negative chronotropic effects and is not contraindicated in heart block or bronchospasm. Clonidine is particularly indicated in severe hypertension associated with pain, anxiety, or withdrawal syndromes, because it has sedative and analgesic-sparing properties [66]. However, it should be avoided in patients with a reduced conscious level. Clonidine should be discontinued gradually because of possible rebound hypertension [67]. In addition, intravenous clonidine can induce a transient increase in arterial pressure so that close monitoring is necessary [68]. Clonidine also can be associated with unpleasant effects, including dry eye and mouth, excessive sedation, and postural hypotension. The availability of other, safer drugs means that clonidine is not used as a first-line agent for severe acute hypertension [69].

##### Dexmedetomidine

Although primarily a sedative agent, dexmedetomidine is a selective α_2_-adrenergic agent with rapid onset of action and easy titration, which could contribute to the control of hypertension in withdrawal syndromes [70].

##### Methyldopa

Initially used in the treatment of eclampsia, intravenous methyldopa is no longer widely used because of its adverse effects. An oral formulation is still available, but the availability of newer drugs with shorter half-lives and fewer adverse effects has limited its use.

### Choosing the most appropriate antihypertensive treatment in the ICU

There is no ideal drug for the treatment of severe acute hypertension in all patients and it is only by recognizing the underlying pathophysiological process and understanding the risk-benefit ratio of each medication that the intensivist will be able to choose the best therapy for the individual patient. A recent report from the European registry for Studying the Treatment of Acute hyperTension (Euro-STAT), including 761 patients treated with intravenous antihypertensive agents in the emergency department, perioperative unit, or ICU, reported that nitroglycerin was the most commonly used antihypertensive treatment (40% of patients), followed by urapidil (21%), clonidine (16%), and furosemide (8%) [8]. However, in a United States registry of 1,588 critically ill patients with acute severe hypertension treated with intravenous therapy, β-blockers were the most frequently used antihypertensive agent (labetalol 32% and metoprolol 17%), followed by nitroglycerin (15%), hydralazine (15%), nicardipine (8%), and sodium nitroprusside (5%) [10]. Interestingly, most of the patients in this survey (64%) required more than one drug for blood pressure control. These regional differences are due to several factors, including marketing influences.

Antihypertensive agents for acutely ill patients should optimally have a rapid onset of action, a short half-life enabling easy titration, and no adverse effects. For the patient with less severe hypertension who will need prolonged treatment, a long-acting antihypertensive may be started at the same time as the short-acting drug.

Some important clinical issues must be considered before starting therapy. First, a careful search and correction of precipitating factors should be made (Table 2). Second, major neurological problems should be excluded. In the presence of intracranial hypertension, every attempt should be made first to control intracranial hypertension before starting antihypertensive drugs, because a reduction in cerebral perfusion pressure (the difference between MAP and intracranial pressure) can precipitate or worsen brain injury. In the presence of intracranial hypertension, vasodilators are not the best agents, because an increase in intracranial blood volume can further increase intracranial pressure. In these circumstances, β-blocking agents are a good option. Third, if drug therapy is needed, it must be based on underlying pathophysiological considerations. For this purpose, one should consider the most important components of acute hypertension: blood volume, heart rate, cardiac function, and vascular tone. Some comorbidities also must be considered: the best example is the cautious use of β-blockers in patients with severe chronic obstructive pulmonary disease or A-V block. Fourth, in all cases, an abrupt reduction in blood pressure must be avoided. In general, the reduction in MAP should not exceed 15-20% in the first 30 to 60 min [27]. A notable exception is the presence of acute aortic dissection for which faster (within 15 min) and greater pressure control may be required [71].

The first question that should then be asked is whether or not cardiac output is increased. Maintenance of a high arterial pressure associated with a hyperkinetic state is only possible when cardiac function is well preserved, because cardiac work (essentially the product of arterial pressure and stroke volume) must be unnecessarily increased. These states are observed when there is hypervolemia and/or increased sympathetic tone. In the presence of hypervolemia, diuretics are the preferred drugs.

In the presence of increased adrenergic tone, tachycardia is typically present. Withdrawal syndromes are an example of this category. β-blocking agents are good options for the control of hypertension associated with hyperadrenergic states. If acute reduction in cardiac output is of concern, a dihydropyridine calcium channel blocker (e.g., nicardipine, clevidipine) may be preferred, because these agents have fewer negative effects on the heart rate and myocardial contractility. These agents provide an interesting option for the treatment of postoperative hypertension [28]. If a vasodilating effect is best avoided (e.g., in the presence of increased intracranial hypertension), clonidine may be a good alternative to β-blocking agents.

If cardiac output is not increased much, then the increase in arterial pressure is primarily the result of increased vascular tone. In these conditions, vasodilators are a logical choice. Venous vasodilators (nitroglycerin) are particularly indicated in the presence of pulmonary edema (in association with diuretics). Nitroprusside remains a valuable agent on a short-term basis in the presence of severe heart failure (because it has no negative inotropic affects). Its balanced effects on the arterial and venous sides of the vasculature make it more effective than nitroglycerin. Its main advantage is its very short half-life, allowing easy titration. Urapidil also may be considered where it is available.

**Figure 1:**
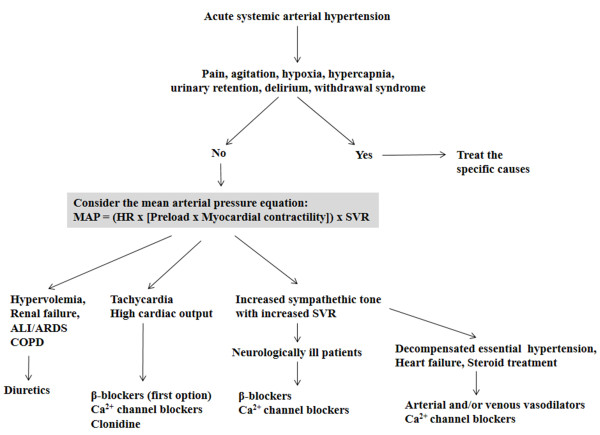
**Pathophysiological approach to the treatment of acute systemic arterial hypertension in critically ill patients based on the main determinants of the mean arterial pressure (MAP).** HR, heart rate; SVR, systemic vascular resistance; ALI, acute lung injury; ARDS, acute respiratory distress syndrome; COPD, chronic obstructive pulmonary disease.

A proposed treatment strategy based on a pathophysiological approach is presented in Figure 1.

## Conclusions

Severe acute hypertension is an interesting hemodynamic challenge. Whereas fluid refractory hypotension is generally treated with a single agent (a vasopressor), hypertension can be treated with a large array of drugs. After recognition and removal of precipitating factors, drug therapy should be initiated and guided by a pathophysiological approach, which consists of the identification of the principal determinants of arterial pressure and a basic pharmacological knowledge of the most frequently used antihypertensive agents. This strategy permits rational management of individual cases and will help to reduce undesirable side effects resulting from inadequate or inappropriate therapy.

## Competing interests

The authors declare that they have no competing interests.

## Author contributions

DRS and JLV drafted the manuscript. DRS, ES, and JLV revised the initial draft. All authors read and approved the final manuscript.
